# Motivations and Limits for COVID-19 Policy Compliance in Germany and Switzerland

**DOI:** 10.34172/ijhpm.2021.30

**Published:** 2021-04-21

**Authors:** Bettina M. Zimmermann, Amelia Fiske, Stuart McLennan, Anna Sierawska, Nora Hangel, Alena Buyx

**Affiliations:** ^1^Institute of History and Ethics in Medicine, Technical University of Munich, Munich, Germany.; ^2^Institute for Biomedical Ethics, University of Basel, Basel, Switzerland.

**Keywords:** COVID-19, Adherence, Germany, Switzerland, Qualitative Research

## Abstract

**Background:** In contrast to neighboring countries, German and Swiss authorities refrained from general curfews during the first pandemic wave in spring 2020, calling for solidarity and personal responsibility instead. Using a qualitative methodology, this study aims to explore why people in Germany and Switzerland were motivated to comply with policy measures during the first wave of the coronavirus disease 2019 (COVID-19) pandemic, and what factors hindered or limited their motivation. While quantitative surveys can measure the level of compliance, or broadly ask what motives people had for compliance, we here strive to explain *why* and *how* these motives lead to compliance.

**Methods:** This publication has been made possible by the joint work of the members of the "Solidarity in times of pandemics" (SolPan) research commons. Seventy-seven semi-structured qualitative interviews were conducted with members of the general public in Germany (n = 46) and the German-speaking part of Switzerland (n = 31) in April 2020. Interviews were transcribed and analyzed following a grounded theory approach.

**Results:** Three themes were identified that summarize factors contributing to compliant or noncompliant behavior. (1) Social cohesion was, on the one hand, an important motivator for compliance, but at the same time related to conflicting needs, illustrating the limits of compliance. (2) Consequences were considered on both the individual level (eg, consequences of individual infection) and societal level (eg, the societal and economic consequences of restrictions). (3) While for some participants following the rules was perceived as a matter of principle, others stressed the importance of making their own risk assessment, which was often associated with with a need for evidence on the effectiveness and reasons behind measures.

**Conclusion:** A variety of motives contribute to COVID-19 related compliance. Authorities should seek to address these multi-faceted aspects to support motivation for compliance in a large proportion of the population.

## Background

**Key Messages**

**Implications for policy makers**

- A sense of togetherness motivates people to comply with restrictive measures and can be fostered through campaigns and shared individual narratives that focus on cohesion rather than division.
- Rules should be consistent, clearly communicated, and easy to follow. This allows people to create new habits and understand the reasons behind restrictions, supporting compliant behavior.
- Providing a comprehensible rationale for the measures implemented may enhance a sense of individual agency to deliberate about personal decisions about the restrictions.
- Communication around the effects and usefulness of restrictive measures can help to reassure people that their individual effort for the broader good is worthwhile.
- Trust in authorities contributes to compliant behavior.

**Implications for the public**

Swiss and German authorities have made recommendations concerning hygiene, social distancing, mask-wearing, and other measures to control the spread of coronavirus disease 2019 (COVID-19), and outlined restrictions that can be tightened once case numbers increase. However, for these measures to be effective, members of the public need to comply with them. Finding ways to foster a sense of togetherness despite mounting societal and economic problems during the pandemic is crucial. When individuals are convinced that their compliant behavior is helpful for the common good, many are willing to make personal sacrifices. At the same time, members of the public have opposing preferences concerning rules and information: While some prefer to have strict rules they can follow, others prefer the space to invoke their own discretion about their personal situation and pandemic recommendations.

When the coronavirus disease 2019 (COVID-19) pandemic first arrived in Europe in Spring 2020, authorities in Germany and Switzerland relied on people’s motivation to comply with public health policies and restrictions. Policy-makers repeatedly and emphatically called for solidarity and personal responsibility, emphasizing that only a joint effort could avoid curfews or other further restrictions.^1-3^ Policy measures were similar in both Germany and Switzerland and included national closures of borders, schools, stores, and businesses. Even though both countries did not evoke general curfews, people were asked to stay home whenever possible and public gatherings were limited to two people in Germany and five people in Switzerland. National face mask obligations were introduced in public transportation and shops in Germany on April 29, 2020,^4^ in Switzerland on July 6, 2020 (public transport only).^5^ A time map of the introduction of policy restrictions has been detailed elsewhere.^6,7^ While in Switzerland some businesses such as hair salons, medical practices, and garden centers reopened on April 27, 2020,^8^ the general easing of restrictions, including the reopening of schools and restaurants, took place on May 11, 2020.^9^ In Germany, some stores and schools were allowed to open again on May 4, 2020.^7^

 This study investigates how and why people complied with restrictions and policy measures during the first pandemic wave in Spring 2020 in Switzerland and Germany, taking an inductive, qualitative approach. We define compliance as a general adherence to the rules and recommendations set out by authorities to protect oneself and others from severe acute respiratory syndrome coronavirus 2 (SARS-CoV-2) infection. While compliance can also be analyzed from a psychological perspective,^10^ we here implement a broader social science approach rooted in the theoretical perspective of solidarity in a public health context.^11,12^ Solidarity in this understanding emphasizes cooperation based upon commonality. It refers to a practice enacted between individuals, within and among groups, but it also can lead to solidified practices with the aim of indirect reciprocity in an institutionalized form. For example, affordable public healthcare is built on reciprocal inter-generational solidarity. By using solidarity descriptively as a practice, we can analyze the norms and motives that accompany compliant and solidaristic behavior.^13^ In this respect, compliance is understood to be solidaristically-motivated if it is perceived as a contribution that benefits others.

 A variety of studies have assessed the level of acceptance and compliance with COVID-19 restrictions. Factors identified as influencing compliance include trust in decision-makers,^14-19^ belief in the effectiveness of measures,^20^ level of information, knowledge, and awareness,^14,21^ perception of risk and illness,^22-24^ perceived self-efficacy,^22,25^ living area,^26^ law enforcement and punishment,^27^ fear of economic consequences,^14,28^ copying the behavior of the social environment,^21,29,30^ and the wish to protect others.^31^ Compliance with social distancing rules was found to be higher in ethnically diverse societies.^32^ A growing number of studies also identify women as being more compliant than men.^19,21,24,33,34^ Longitudinal studies in Spring 2020 found a decrease in compliance over time in Norway and Germany.^7,35^ However, most existing research have been quantitative studies based on survey data^7,14,18,20-23,25,29,33,36^ or mobility data based on digital monitoring.^15,17,26,32,37^ Only a handful of qualitative studies exist so far on public compliance with COVID-19 restrictions: Ölcer et al^28^ analyzed social media posts qualitatively to assess the lay perspectives on governmental intervention in the COVID-19 pandemic, finding that misinformation, uncertainties related to the pandemic, and the important influence on individual social life limited compliance. Wong et al^16^ found through the analysis of social media posts and online focus groups in Singapore that high trust in authorities led people to underestimate the risks of the COVID-19 pandemic, leading to lower compliance. Williams et al^31^ assessed perceptions of social distancing in UK-based focus groups with laypersons, identifying a high motivation to protect the vulnerable for social reasons, despite lack of trust in the government. Moreover, some studies analyzing compliance with mask-wearing relied on observational data.^27,38^

 Survey studies from Germany and Switzerland reported a high level of trust in government during the first restriction period in Spring 2020.^20,39-43^ For example, more than 80% of surveyed individuals in Germany reported that they had limited their social contacts, avoided traveling, and washed their hands more often than usual.^20^ Nonetheless, longitudinal data indicated a steady decline in support for the restrictive policies during April and May in both countries.^7,41-43^ For example, more than 50% of German participants supported stay-at-home orders end of March, whereas in May it was only 10%. Closing public facilities and borders were supported by more than 90% in March, but support declined to 40% and 60%, respectively, in May.^7^

 While quantitative studies can measure the level of compliance, or broadly ask what motives people had for compliance, they are not able to explain *why* and *how* these motives lead to compliance. Therefore, and in contrast to previous studies, compliance is investigated using a qualitative methodology to understand the motivations and limits to policy compliance. To our knowledge, this is the first study using qualitative one-to-one interviews with members of the general population to address this question. This publication has been made possible by the joint work of the members of the “Solidarity in times of pandemics” (SolPan) research commons.^44^

## Methods

 Semi-structured interviews with inhabitants in Germany and the German-speaking part of Switzerland were conducted as part of a qualitative, longitudinal, and multinational research study in collaboration with the SolPan research commons.^44,45^ SolPan’s aim and interview guide were informed by the theoretical framework of solidarity as proposed by Prainsack and Buyx.^11,12^ The interviews did not focus on solidarity specifically but asked about interviewees’ experiences during the pandemic, as well as their motivations for complying with COVID-19 related public health policies during the first pandemic wave in April 2020.^46^

 This study includes the views from German and German-speaking Swiss inhabitants because both countries have managed to flatten the first curve of new infections effectively with less strict measures than other European countries such as Italy at the time,^20^ with a strong narrative of policy-makers calling for people’s solidarity and responsibility. Including data from two separate countries allows for a higher variety of perspectives, which is one of the strengths of qualitative studies.^47^ The study allows us to understand motives leading to compliance or noncompliance, provide context for decisions relating to compliance, and discuss the meaning of specific practices in the context of the pandemic.

 The Consolidated Criteria For Reporting Qualitative Research (COREQ) checklist^48^ was used as a reporting guideline (Supplementary file 1).

###  Recruitment and Data Collection

**Figure 1:**
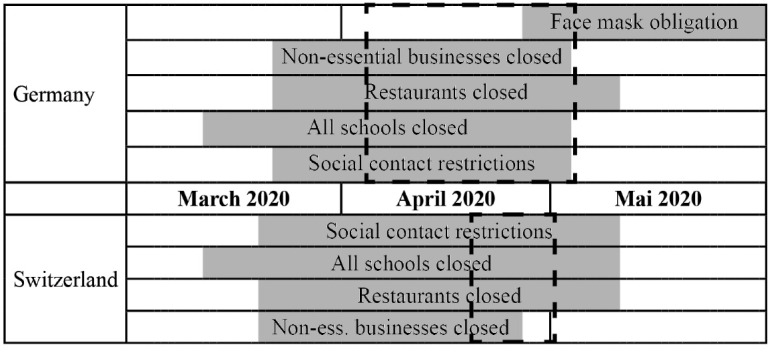
Overview of National Restrictions During the First COVID-19 Pandemic Wave in Spring 2020. Please note that due to the federalist organization of both countries, certain regions had invoked restrictions earlier. The dashed squares correspond to the time of data collection. Abbreviation: COVID-19, coronavirus disease 2019.

 Participants from Germany (n = 46) and German-speaking Switzerland (n = 31) were recruited through online advertisement on the University websites and social media networks of the institutions collaborating in the SolPan research commons, convenience sampling, and snowballing. Most individuals interested in participating contacted the research team directly, which is why no reliable dropout rates are available. Some individuals proactively asked for an interview via snowballing later declined due to time constraints. Each participant gave one interview between April 6 and May 6, 2020 (Germany) and between April 19 and May 1, 2020 (Switzerland). Figure 1 indicates the time window of data collection relative to national restriction policies.

 The recruitment strategy was aimed to capture a broad variety of perspectives within one country in a particular time frame. This strategy was chosen to account for the rapid evolution of the pandemic and the related policy landscape. To enable a maximum variety of perspectives, participants were recruited with different demographics, including age, gender, income, household structure, living area (rural – town – city), education, and employment situation (Table).

**Table:** Demographic Distribution of Participants

**Category**	**Germany**	**Switzerland**
Age		
18-30	9 (19.6%)	8 (25.8%)
31-45	19 (41.3%)	6 (19.4%)
46-60	5 (10.9%)	7 (22.6%)
61-70	8 (17.4%)	5 (16.1%)
70+	5 (10.9%)	5 (16.1%)
Gender		
Female	24 (52.2%)	16 (51.6%)
Male	22 (47.8%)	15 (48.4%)
Household		
Single	13 (28.3%)	8 (25.8%)
Couple	16 (34.8%)	10 (32.3%)
Living with child/children under 12	8 (17.4%)	3 (9.7%)
Living with child/children 12+	4 (8.7%)	5 (16.1%)
other	5 (10.9%)	5 (16.1%)
Rural/urban		
Big town^a^	22 (47.8%)	10 (32.3%)
Medium/small town	12 (26.1%)	6 (19.4%)
Rural (eg, village)	12 (26.1%)	15 (48.4%)
Employment status		
Employed (long-term contract)	21 (45.7%)	13 (41.9%)
Self-employed	4 (8.7%)	3 (9.7%)
Employed (short-term/precarious contract)	3 (6.5%)	6 (19.4%)
Unemployed	4 (8.7%)	1 (3.2%)
Retired	10 (21.7%)	7 (22.6%)
other	4 (8.7%)	1 (3.2%)
Education level		
Less than 10 years	2 (4.3%)	10 (32.2%)
10-14 years (eg, high school diploma)	16 (34.8%)	3 (9.7%)
Higher education	28 (60.9%)	18 (58.1%)
Household net income		
Up to 1400€ (4000CHF)/month	5 (10.9%)	6 (19.4%)
1401-3000€ (4001-7000CHF)/month	14 (30.4%)	9 (29.0%)
More than 3000€ (7000CHF)/month	27 (58.7%)	16 (51.6%)
Total	**46**	**31**

^a^Defined as more than 500 000 inhabitants (Germany); or more than 100 000 inhabitans (Switzerland).

 Interviews ranged from 30 to 45 minutes and were conducted online or by phone (see Supplementary file 2 for author contributions and qualifications). One interview with a German resident was held in English, the rest were in German. Most participants were alone during the interview, but in some cases family members (spouses or children) were present. Those circumstances were noted in field notes by the interviewer. The interviewer was always alone when conducting the interview. Interviewers and participants did not know each other personally.

 Before the interview, participants received in-depth information about the study and any questions they had were answered. Consent was obtained orally directly before the interview. The consent process and the subsequent interview were recorded on a digital recorder or using a General Data Protection Regulation-compliant video chat recorder (eg, GoToMeeting). Only audio, not video material was stored for transcription, and transcripts were pseudonymized. Swiss interviews were held in Swiss German dialect and translated to standard German upon transcription. Transcripts were not returned to participants.

###  Data Analysis

 The SolPan research commons generally follows a constructivist grounded theory approach^49^ that has been adapted for large-scale qualitative comparative research (a detailed research protocol is currently in review).

 First, using Atlas.ti 8.0, all interviews were coded using an inductively generated Master Coding Scheme developed by the SolPan data analysis group.^50^ This made data accessible for content-specific analytical work and helped the researchers to familiarize themselves with the data. The coding of each interview was checked by a second researcher for consistency.

 Second, relevant text passages concerning compliance were extracted using the Atlas.ti query function and analyzed inductively, looking for emerging themes and relationships (the queries are presented in Supplementary file 2). Interviews from each country were first analyzed separately and then compared, combined, and contrasted in team discussions. Through an iterative process, which was characterized by a continual comparison between interviews, countries, and memos, we condensed the findings into the three themes presented below. Each analytical step was accompanied by extensive discussions in the research team. We refrained from sending these findings out to participants for feedback at this stage because we conduct several topic-specific analyses and do not consider it appropriate to constantly contact them. However, findings were reviewed and additionally analyzed based on the feedback of five independent reviewers.

 Following a pragmatic definition of data saturation,^51^ we addressed data saturation through analytical rigor and by aiming for a maximum variety of perspectives when recruiting a variety of demographics.

## Results

**Figure 2:**
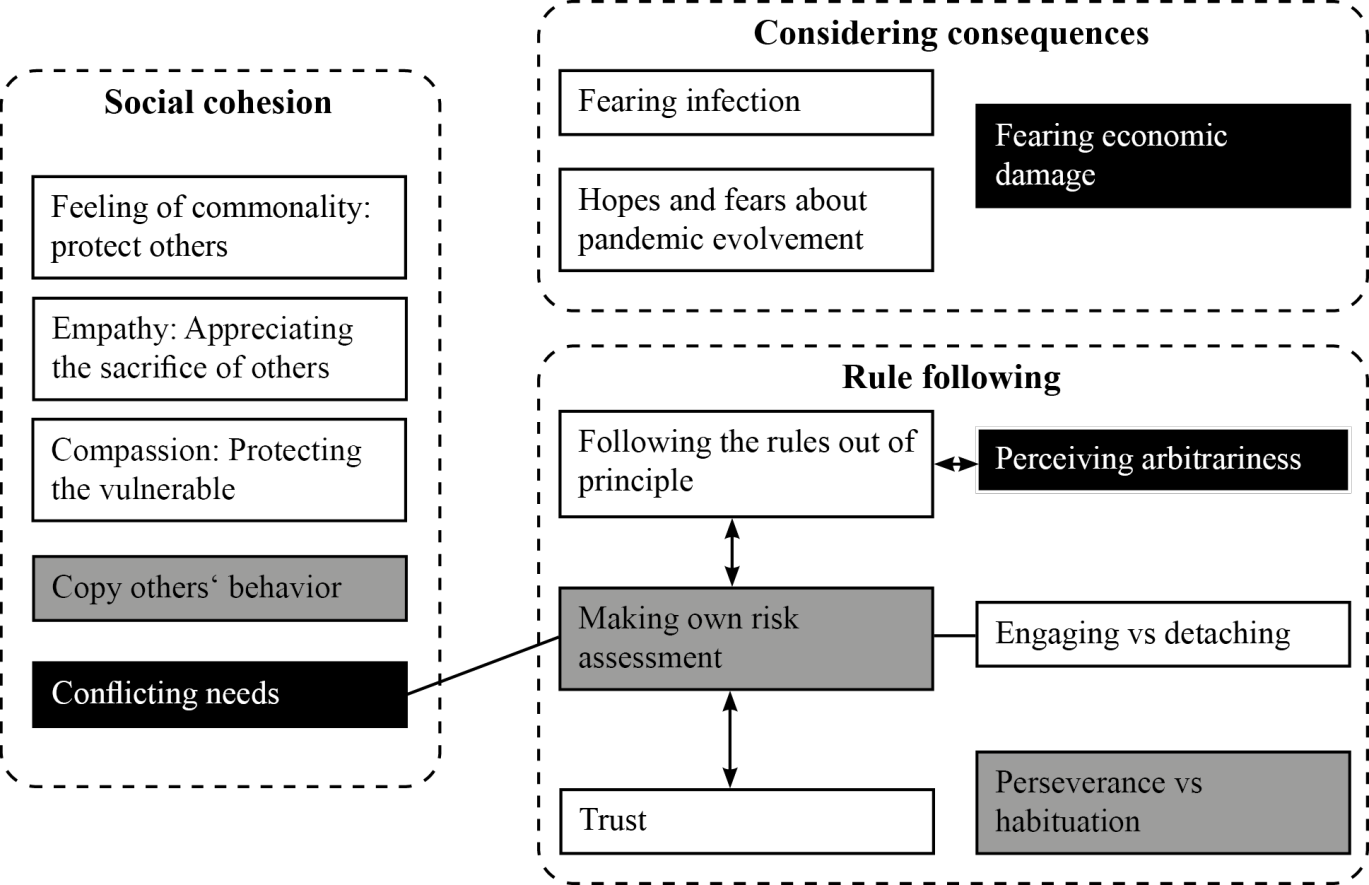
Overview of Themes Summarizing Motivations and Hindering Factors for Compliance With COVID-19 Related Public Health Policies During the First Pandemic Wave. White boxes = motivating factors; black boxes = hindering factors; grey boxes = can be motivating or hindering. Abbreviation: COVID-19, coronavirus disease 2019.

 Three themes were identified from the interview data that summarize motivating and hindering factors for compliance with COVID-19 related public health policies during the first pandemic wave in Germany and Switzerland: (1) Social cohesion; (2) Considering consequences; (3) Rule following. Figure 2 illustrates these themes and what they entail. There are important differences between individual participants concerning the importance of each aspect; some mentioned one dominant factor that motivated them to comply (or not); others mentioned a variety of influencing factors. Quoted participants are anonymous, but each participant received a country code and a number that are provided after each direct quotation (eg, “DE11” stands for German participant number 11; “CH09” for Swiss participant number 9).

###  Social Cohesion

####  Feeling of Commonality

 One motivation for compliance was connected to a sense of togetherness which was based on a feeling of commonality. Participants were motivated to comply to protect others from infection, which points to a feeling of connectedness among society. Some referred to a feeling of responsibility towards their social environment and felt that everybody’s help was needed to limit the spread of the virus. Accordingly, not complying with social distancing rules was called irresponsible and egoistic by some participants. Others stated that they complied because they wanted to set a good example for others and acknowledged the force of social cohesion.

 Observations that other people were not complying with the guidelines sparked feelings of anger among those who had been making efforts to comply. Noncompliance was perceived as “ruthless,” as it would put everybody in danger. Seeing groups of younger people outside during springtime was met by feelings that they were being irresponsible or selfish, or that they did not understand the potential consequences of the virus for other, more vulnerable groups. Adding another perspective, one participant described overhearing an individual at the supermarket making plans on the phone for a private party involving alcohol. Overhearing the conversation, the participant described their irritation:

*“I turned around and said: Excuse me, but you still haven’t gotten it yet? I was so pissed off, I wanted to tell him, you know, I mean, I’m 50+. Among other things, we don’t just do this because of the old people, but also because of you guys. Not because you could fall seriously sick, but because you want to go back to your festivals and you want to go back to your clubs and dance”* (DE11).

####  Empathy and Compassion

 Participants described the necessity to protect the vulnerable. This was framed especially as a kind of intergenerational solidarity: the younger support and protect the elderly by complying with the rules. Protecting the vulnerable, some said, such as elderly individuals, requires younger individuals to work together and create community resources for doing shopping or distributing things. However, the motivation to protect the vulnerable was repeatedly connected to high expectations of the elderly staying at home, with some expressing astonishment or anger if the elderly were not complying. Others observed teenagers and young adults sitting together in larger groups.

 In addition, a couple of participants expressed appreciation for others’ sacrifices as a motivator for compliance. For example, when hearing of other difficult situations, such as the spike in cases in New York City in April 2020, some participants expressed a need to act in solidarity with others who were “on the front lines” fighting the virus, such as healthcare workers: “*That’s why I think it’s so important that we who may not be on the front line show solidarity and just stay at home and not take any additional risk in order not to overload the system even more. I fully agree that this is something we should adhere to*” (CH09).

####  Copying Others’ Behavior

 Another form of social relatedness was illustrated when a few participants described how they took orientation from the behavior of others. For example, some participants stated that they would wear face masks if everybody would. Others described how they saw people in the park and on the playgrounds, awakening their desire to go there, too. Thus, the social behavior that people were surrounded by seemed to influence their own.

####  Conflicting Needs

 While the feeling of togetherness and commonality served as an important motivator for compliance, it was also an important hindering factor. First, some participants expressed a feeling of loneliness and isolation, which led them to make exceptions to the guidelines.

*“The biggest challenge is to carry this through in a really consistent way. I already broke the rules two or three times. Last Saturday, for example, I had a strange feeling, because you know it’s better not to do it. But at some point, you feel really cooped in and then even an online beer with a friend is not enough. […] And then we sat there, the four of us” *(DE03).

 Second, the desire to protect the vulnerable from infection sometimes conflicted with other needs of the vulnerable. For instance, some participants noted that face masks could alienate small children or people with dementia or lead to social isolation and depression of the elderly. For some, not seeing family members was worse than the risk that came with contracting the virus:

*“And my [mother] is very clear about that. She says, ‘I am 84 years old. So if this virus kills me now, well, so be it, but I think it’s much worse that you won’t visit anymore.’ And I have had the same experience with the old people in the elderly home. Many people there very clearly say: This restriction that they can’t see their children and grandchildren is worse than taking the risk of simply dying from this virus. Those who are really old and have made their peace with it. I think that is somehow overlooked”* (DE11).

 Participants also reflected on differing needs concerning children and explained why they made exceptions based on individual needs. One parent described how their 9-year-old son had suffered a lot from following social distancing guidelines and they decided to let him play soccer in the backyard with one other child, as long as they did not touch each other. As a reaction to such conflicts, many participants explained how they found compromises to satisfy both their social needs and the social distancing rules. We introduce and discuss this aspect in more detail in the subsection “Personal risk assessment.”

###  Considering Consequences

####  Fearing Infection

 Many participants stated that they complied because they wanted to protect themselves and others from infection, indicating their interest in taking responsibility for their own health but also for others.

 Some participants were very afraid of getting infected; protecting themselves was their main motivation for compliance. This was particularly true for those who considered themselves to be particularly at risk for COVID-19-related complications due to chronic illnesses or age, but it also applied to few participants who did not belong to any official risk group. The perception of being at risk was highly individual:

 “*In the beginning, I was surprised that many people have the feeling: ‘It doesn’t affect me at all, I can keep doing things as always.’ Even older people, my own parents, but also my husband’s parents, who thought they could go shopping every other day. That might have taken a little time for them to get used to or to handle things differently*” (DE29).

####  Hopes and Fears About Pandemic Evolution 

 Many participants were motivated by the perception of being able to act in a way that helps limit the pandemic. For example, they hoped for a faster easing of restrictions if everybody complied with policies. The fear of negative consequences, of making things worse, was an additional driver of compliance. Participants feared a second infection wave as “the worst-case scenario” (CH13) or expressed worries of having to begin a full lockdown, appreciating the remaining freedom to go outside, noting that “we are not over the worst yet” (CH03). For many participants, individual behavior was a critical factor in the development of the pandemic.

####  Fearing Economic Consequences

 Fears of the economic consequences limited compliance. Several participants referred to the perceived dilemma between public health protection and economic survival, noting that there were likely to be huge economic damages, the extent of which were not yet known, but that ultimately it was a matter of life in death. In this moment of crisis, one participant noted, health must come first. Others described economic concerns such as high unemployment and rising debt but noted that it was not yet possible to go “full throttle” economically and return to business as normal when the threat of a second-wave loomed. These dilemmas threw into sharp relief the costs of re-opening the economy, and the acknowledgment that lives would be lost:

*“I can understand it, and it is always a matter of weighing how much money a human life is worth. I think that is the basic question that we need to ask ourselves at the moment: How much is a human life worth compared to the survival of a company? But I have many friends whose companies are bankrupt or who are unemployed. Or, in the best case, are on 40% short-time work. In this respect, I can understand their concerns”* (CH16).

###  Rule Following

 This theme explores the tension between following rules imposed by authorities out of a sense of duty and obligation, versus the wish for personal risk assessments and discretion.

####  Following Rules Out of Principle

 For some participants, compliance was a matter of principle: “*We actually follow everything. We are such a rule-obsessed family, including my children*” (DE07). They felt that complying with policy measures was the right thing to do both in public and private. For example, even though some expressed reluctance to wear face masks and doubted their efficacy they still wore the mask regardless when in public if it was the rule: “*I put on the mask because it is simply required. But I’m not really sure if it is of any use. Actually, I only do it because it is required by law*” (DE15). In those instances, rule-following trumped the wish to understand the reasons behind measures.

####  Personal Risk Assessment

 By contrast, other participants stressed their willingness and the importance of assessing their personal risk rather than blindly following rules, detaching themselves from what they perceived as the rule-obeying majority of the population: “*If our federal government would say it helps against COVID-19 to hit yourself on the head three times with a stick in the morning, I think eighty percent would do it, jokingly speaking*” (DE20).

 For those participants, understanding the reasons for a given guideline was crucial for compliance. Some also expressed a wish for evidence concerning the effectiveness of measures, and were frustrated by the absence of it: “*So at the moment it’s just the stupid mask question. You don’t know, does it do anything or does it not?*” (CH03).

 Some participants described how they calculated their decision to comply based on their personal risk assessment and defined their own behavioral rules. While for some this behavior was linked to a general desire for autonomy and personal responsibility, it was often additionally motivated by the wish for social contacts, illustrating, as outlined above, how social cohesion served as both a motivating and hindering factor for compliance. Additionally, some individuals stressed the difference between mandatory measures and recommendations to justify their behavior:

*“We made one exception at Easter when my parents-in-law came for a visit. We were sitting in our garden and just celebrated Easter with them. But kept our distance. And my brother with his girlfriend, they came the next day, and we did it the same way. Yes, there we might have – or no, we didn’t actually break the rules. But they recommended that people should stay at home and not meet each other. But, yes, they visited us, but with distance”* (CH28).

 Perceived arbitrariness of policy measures limited compliance for these individuals and led to irritations. One participant, for example, was asked to take a shopping cart before entering the store as a means of controlling the number of shoppers. Given that the participant only wanted to buy bread at the bakery, which was located immediately beyond the entrance, the participant was irritated by the lack of discretion in the guidelines. Participants sometimes doubted the reasonableness and proportionality of particular measures, thinking that they might cause different problems that might weigh even more heavily than the actual disease.

####  Engaging vs Detaching

 Some participants expressed the need and the motivation to take responsibility for themselves and their life in the pandemic, which included proactively seeking out information to comply with restrictions and organizing their lives following those restrictions. The wish to understand the reasons for measures reflected an approach of some participants to take control of an uncertain situation.

 “*To reopen my business later, […] I have to find out what the protection orders are that I have to address. How do I need to do that? And people have to compile a lot of information themselves. For example, how to reopen their businesses again. Because nobody comes along and says, listen, you are going to open your [business] again, you have to do this and that. Instead, I have been increasingly noticing that people have to inform themselves to do it right”* (CH14).

 Accordingly, those participants complied better with restrictions if they understood and accepted the risks of the viral spread and the potential costs concerning public health and personal well-being. Others, by contrast, needed personal detachment to better cope with the unprecedented situation, as it helped them accept the collective incertitude:

*“I have tried not to let Corona get too close to me anymore, and to occupy myself with other things. This fear is coupled with this crazy helplessness, that we have actually no idea what we can do about [the pandemic]. There is really nothing we can do, except to obediently stay at home”* (DE16).

####  Trust

 Another motivation for compliance with restrictive public health policy was having trust in authorities, including the government and scientific experts. One participant noted that they preferred to have guidance from authorities, and intended to follow those instructions: “*The whole situation is still so opaque that I prefer this being controlled from above. Not that I want to give up the responsibility to think, but I simply have confidence in those who are responsible now, and I obediently follow these instructions*” (CH14).

 Trust also promoted compliance in participants who felt overwhelmed by the situation and were unable to make their personal risk assessment:

*“Yes. I kind of have to trust, let’s say: I just don’t know any better. And I don’t have serious cause to doubt. And I know that all politicians are human beings as well. And all of them have to rely on the expertise of specialists. […] It just costs me additional energy to doubt, whether the regulations are right, and I hope to contribute well to get this over with soon”* (DE05).

####  Perseverance vs Habituation

 Several participants described their impression that rule-following became more difficult over time and that they felt that after a couple of weeks people became more indifferent to the restrictions. For example, one elderly participant stated:

*“Some of my friends were […] really stubborn and strict at the beginning and did not want to leave the house and were almost hysterical. But now, since one or two weeks, they say ‘I can’t stand it anymore. I go out, I just do it.’ And I just found out that they go grocery shopping every now and then. […] So now people are becoming indifferent and that’s not good, I think. We should stay patient, now that we have lasted so long already” *(CH23).

 In contrast, other participants felt that rule-following was becoming easier over time, as the changes in routine were becoming a habit. Eventually, they noted, following increased hygiene measures or paying attention to physical distancing might simply become second-nature when interacting with strangers: “*At some point, they become processes that we don’t even notice anymore. And then you just do it*” (CH30).

####  Country-Specific Differences

 We observed that German participants tended to focus more on the rule-following aspect whereas Swiss participants tended to emphasize the need for their personal risk assessment. There are counterexamples in both cohorts, but the general tendency leads us to hypothesize a potential difference in compliance, which will be discussed further in the discussion section.

## Discussion

 This qualitative interview study with German and Swiss inhabitants explored what motivated people living in Germany and the German-speaking part of Switzerland to comply with policy measures during the first pandemic wave of COVID-19. We found that a variety of individual motives influenced people’s compliance.

###  Solidarity and Personal Responsibility

 Some participants were motivated to comply out of a feeling of the common good and to protect others, seeing compliance as an act of solidarity. We extrapolate from our findings that policy-maker’s public appeals to solidarity during the first pandemic wave^1-3,52^ have proven to have been grounded in people’s realities and addressed prevalent, pro-social motives. At the same time, participants yearned for social contacts to maintain a sense of togetherness and to avoid feeling isolated and lonely. This need illustrates the limits of solidaristically-motivated compliance and connects to survey studies indicating that small social networks led to higher fatigue^53^ and that a perceived threat to one’s socio-cultural identity led to less adherence.^54^ Participants also reported what we interpret as early disturbances in a feeling of togetherness, especially in cases of intergenerational conflicts where the elderly accused the younger generation and the younger the older generation of not complying sufficiently. Even though the importance of such intergenerational solidarity has been repeatedly stressed in the context of the COVID-19 pandemic,^55,56^ this example illustrates that solidarity during the COVID-19 pandemic cannot be taken as a given.^12,57^ Our findings also lend some support to public bodies that warned early on that the motivational ‘resources of solidarity’ were not bottomless.^58^

 Alongside calls for solidarity, personal responsibility has been an often-used rhetorical tool in Swiss and German public debates in the context of the COVID-19 pandemic.^59,60^ Indeed, our findings also indicate a deep sense of responsibility towards oneself and others among our participants, which to many also increased perceptions of control and agency. This was accompanied by a high perception of self-efficacy,^61^ which has been identified as an important factor for compliance in survey-based studies.^14,25^ To enable people to take personal responsibility requires transparent communication so that people understand the reasons for measures. Otherwise, some people may be reluctant to comply with the imposed guidelines because they lose trust in the meaningfulness of their personal sacrifice.

 As stressed by our participants, it also becomes more difficult with time to maintain the discipline necessary to stick to the public health policies out of personal responsibility, as also shown in a German longitudinal survey.^7^ Therefore, the longer the pandemic goes on, the higher the personal costs of compliance are, and the fewer policy-makers can rely on personal responsibility and solidarity as the main motivators for compliance.^12^

 Moreover, the fact that Germany and the German-speaking part of Switzerland were not as heavily affected as other parts of Europe during the first pandemic wave^62^ could make people unwilling to repeat such personal efforts in further pandemic waves due to the so-called ‘prevention paradox’^63^– because measures that were effective and lead to flatter pandemic curves are paradoxically seen as having been unnecessary.

###  Coping With Uncertainty

 Some participants seemed to find compliance to be a helpful psychological coping mechanism, as it gave them some guidance on how to behave in such a new situation that was full of uncertainty. Supporting this, a survey study from Cyprus found that individuals illustrating higher compliance with precautionary measures tended to report lower depression levels.^34^ Several factors set out to explain this: The rapid spread of SARS-CoV-2 over the European continent came unexpected for policy-makers and the general public alike, and risk perception increased importantly in a short period,^64^ which explains the perceived behavioral uncertainty among participants. Moreover, human beings have difficulties in assessing risks related to exponential growth^65^ which is necessary to understand the necessity of restrictive measures in a pandemic.^66^

###  Trust

 Trust in authorities has already been identified in previous surveys and inquiries as an important compliance factor.^15,17-19^ Because trust in government is generally high in Germany and Switzerland as compared to other countries^67^ it has already been speculated to be one of the reasons why Germany and Switzerland did comparatively well in the first pandemic wave.^62^ Indeed, trust in authorities has been generally high in Switzerland and Germany in the first pandemic wave^68,69^ and, accordingly, most of our participants expressed a high level of trust towards public health officials. However, while trust in authorities made it easier to accept existing rules and restrictions, it was not the only motivation for compliance. This is also supported by a UK-based qualitative focus group study, where people complied despite a lack of trust in the UK government because they wanted to protect the vulnerable.^31^

 Some studies indicate that countries with a higher “social capital,” including trust, social norms, and social networks, tend to have better compliance and lower infection rates when investigating over a longer period,^18,70^ and these factors were especially important in the absence of strict policies.^71^ This further supports the notion that trust in authorities plays a part in people’s compliant behavior, but other factors, such as social cohesion, are needed as well.

###  Noncompliance

 All identified themes can serve as motivators to comply with public health policies but are also instructive in considering non-compliance. If public trust in authorities erodes and people lack understanding and acceptance of policy measures, they become increasingly indifferent in complying with policies. At the same time, following rules and taking personal responsibility requires discipline which becomes increasingly difficult to maintain with time due to an increased perception of the personal costs. Thus, a fine balance between legal reinforcement and punishment, information campaigns, and space for freedom and liberty to take personal responsibility is required to encourage continued compliance.

 Moreover, if people do not have the necessary support systems, such as childcare or social support for the elderly, they will have more difficulties complying with the guidelines. Suitable childcare facilities and economic support systems are, therefore, not only important to limit the economic damage invoked by policy measures, but also enable people to stay compliant with public health policies.

###  Country-Specific Contexts

 We reported the observation that Swiss participants tended to value personal responsibility more than German participants and, by contrast, German participants tended to focus more on rule-following out of principle and references to discipline. These observations should be interpreted as a hypothesis rather than a finding due to the qualitative nature of this inquiry. We now aim to test and contextualize this hypothesis.

 First, in terms of culture, the Hofstede model indicates that the German culture has a higher need for uncertainty avoidance, a stronger focus on long-term orientation, and tends to be more restrained than the Swiss culture.^72^ The observed tendencies in the data are in line with the Hofstede model: Swiss participants tended to prefer finding compromises to fulfill both their personal needs and policy restrictions, establishing their own boundaries of what they perceived as being compliant with policy measures. They wanted to understand the reasons for policies and expressed a strong drive to take personal responsibility, whereas German participants tended to focus more on rule-following and discipline, which would be in line with the more uncertainty avoidant and restraint German culture. As a limitation, the Hofstede model only reports Swiss culture as a whole and does not distinguish between language regions, which are known to be different.^37^

 Second, while Germany and Switzerland invoked similar policies in response to the first pandemic wave in spring 2020, policy strategies started differing significantly in response to the second pandemic wave. Germany invoked new rules and fines quite early on. The limitation of private social gatherings and closure of bars and clubs were generally well supported by the public, although restaurant closures were rejected by the majority of survey respondents.^50^ By contrast, Swiss policies strongly relied on personal responsibility and lose restrictions despite comparatively high case numbers. While restrictions were well-supported by a large majority of people, around half of the survey participants were in favor of even stronger restrictions, such as a short lockdown.^51^ The observed tendencies of stressing personal responsibility reflect these different policy strategies.

 Third, in terms of political systems, both countries are federalist democracies with neo-corporatist features. Switzerland is a direct democracy; the people are the sovereign. Germany, by contrast, is a representative democracy with the parliament representing the people. The systemic differences that make Swiss people accustomed to direct political responsibility also speak in favor of the observed differences. Moreover, economic associations and interest groups are highly influential in Swiss policy-making; more so than in Germany.^73^ They are expected to have an increasing influence on policy-makers particularly as the COVID-19 crisis leaves its initial emergency state after the first pandemic wave.^6^

 Still, the extent and importance of these differences need to be assessed in a quantitative inquiry in future studies. From an international perspective, it illustrates that context matters; cultural and political identities might lead to differing reactions, making country-specific or even region-specific regulations and restrictions valid and necessary.

###  Limitations

 This is a qualitative study, we do not aim for representativeness or statistical significance but theoretical saturation and completeness. Recruitment of participants was restricted due to external circumstances; the SolPan commons was set up on short notice in response to the rapid development of the COVID-19 pandemic in March 2020 and we sought to recruit and interview participants while lockdown restrictions were still in place. This limited the time window for the interviews to April 2020, which placed constraints on the assessment of theoretical saturation based on the content of interviews. To that extent, one limitation is that, even though we have sought out a variety of perspectives through demographic variation, our sample did not at the time contain any explicit ‘COVID-19 deniers,’ although we did have participants who emphasized that it was comparable to the flu. At the time of the investigation, individuals with ‘denial’ attitudes or supporters of ‘COVID-19 conspiracy theories’ attitudes were the absolute minority in both countries according to surveys and most people accepted that some restrictions were necessary.^74,75^

 Moreover, even though participants covered a broad spectrum of demographics (Table), the majority of participants had a high income, higher education, and long-term employment. Due to the low numbers of participants with precarious contracts or unemployment, the theoretical saturation of this group might be not as strong as for other employment groups. It is also generally possible that people who were under particular pressure during the lockdown, for example, single parents or those not coping well with the restrictions, were underrepresented as they did not find the time and energy for an interview. Thus the motivations and limits for COVID-19 policy compliance among those in precarious living situations require further investigation. In Switzerland, we only interviewed inhabitants from the German-speaking area. Because there are considerable cultural differences between the Swiss language regions that led to different motivations and attitudes towards public health policies,^37,76^ these results do not necessarily refer to the views from the French- and Italian-speaking regions of Switzerland.

 This study did not aim to analyze motivations and limits for complying with specific measures, nor did we distinguish between different types of compliance. Due to the explorative qualitative study design, we instead aimed to probe compliance during the lockdown period, contributing a dataset that complements existing quantitative literature on more specific aspects of compliance.

## Conclusion

 This qualitative study contextualizes and connects a variety of factors leading to non-compliance, thereby supporting the numerous quantitative surveys that have been conducted worldwide about motives for compliances.^7,14,18,20-23,29,33,36,39,40^ This highlights why qualitative work is essential to understand how motivations affect and influence behaviors. The results can help to design policy measures during times of a pandemic that can be designed more effectively and more sensitively.

 Policy-makers need to be aware that for compliance with pandemic measures that deeply impact people’s lives, there are a variety of motives that exist simultaneously. This needs to be taken into account when communicating policy measures; appealing to a variety of motives could lead to better overall acceptance and compliance than general appeals to solidarity or rule-following to tackle the crisis together. While we find that solidarity can be a supportive factor for compliant behavior, a policy strategy should not rely too heavily or exclusively on solidaristic behavior, because individual sacrifices increase over time and this brings solidarity to its limits. Moreover, social distancing measures act against a sense of togetherness that assists solidarity-based compliant behavior. Consequently, there could be a danger that solidarity as a motivational factor gets ‘depleted.’^58^

 Consistent and clearly communicated rules that are easy to follow, such as frequent hand-washing, are important to decrease uncertainty and maintain people’s perception of agency. They allow for new habits that support people in complying with measures. The confusion of mask-wearing expressed by participants before mask obligations were introduced provides a counterexample, as rules were not clear and consistent. Even though wearing a face mask is not a particularly complicated action to comply with, people expressed uncertainty about when to wear one. Those who perceive the pandemic as particularly threatening and distressing use authoritative rules as an orientation that also supports their coping behavior and resilience. At the same time, however, others emphasize personal responsibility and freedom: they need space for self-interpretation and individualized ways to protect themselves and others. Depending on culture, pandemic situations, political systems, and healthcare systems, policy-makers need to find a balance between restrictions and recommendations that are adapted to the particular context.

 Our findings also support the great importance of transparent communication. Authorities should provide a comprehensible rationale for measures invoked and should also monitor and communicate the effects of particular policies. This not only increases trust in authorities, which is a crucial prerequisite for compliant behavior in our study, but also increases the perceived efficacy of personal sacrifice and allows people to take personal responsibility.

## Acknowledgments

 The data for this study was collected as part of the SolPan research study, which was conducted by the SolPan research commons. The SolPan research commons has conceptualized and designed the study, including the interview guide and the master coding scheme used for data analysis. We also thank Eric Paul, Paul Stephan and Magnus Tibbe for initial data coding and assistance.

## Ethical issues

 The study was approved by the Technical University of Munich’s ethics committee (no 208/20 S).

## Competing interests

 Authors declare that they have no competing interests.

## Authors’ contributions

 Conception and design: AB, BZ, NH, and SM; Acquisition of data: AB, AF, AS, BZ, and NH; Analysis and interpretation of data: AF, BZ, NH, and SM; Drafting of the manuscript: BZ; Critical revision of the manuscript for important intellectual content: AB, AS, AF, NH, and SM; Obtaining funding: AB with support from AF, AS, BZ, NH, and SM; Supervision: AB.

## Funding

 This work was supported by the Bundesministerium für Bildung und Forschung [Grant number 01Kl20510].

## Supplementary files

Supplementary file 1. COREQ (Consolidated Criteria for Reporting Qualitative Research) Checklist.

Supplementary file 2. Methods Additional Information.
