# Supplementary material for: Motivations and Limits for COVID-19 Policy Compliance in Germany and Switzerland
Source: Int J Health Policy Manag. 2021 Apr 21;11(8):1342–53. doi: 10.34172/ijhpm.2021.30 (PMC9808338; doi:10.34172/ijhpm.2021.30)
Supplement: Supplementary file 1 — COREQ (Consolidated Criteria for Reporting Qualitative Research) Checklist. [file ijhpm-11-1342-s001.pdf]

Article title: Motivations and Limits for COVID-19 Policy Compliance in Germany and Switzerland

Journal name: International Journal of Health Policy and Management (IJHPM)

Authors' information: Bettina M. Zimmermann[1,2]*, Amelia Fiske[1], Stuart McLennan[1,2], Anna Sierawska[1], Nora Hangel[1]¶, Alena Buyx1¶

[1]Institute of History and Ethics in Medicine, Technical University of Munich, Munich, Germany.

[2]Institute for Biomedical Ethics, University of Basel, Basel, Switzerland.

(*corresponding author: bettina.zimmermann@tum.de)

¶ Authors 5 and 6 contributed equally to this paper and share last authorship.

Supplementary file 1: COREQ (Consolidated criteria for Reporting Qualitative research) Checklist

Topic	Item No.	Guide Questions/Description	Reported on Page No.
**Domain 1: Research team and reflexivity**			
Personal characteristics			
Interviewer/facilitator	1	Which author/s conducted the interview or focus group?	Supplementary File 2
Credentials	2	What were the researcher's credentials? E.g. PhD, MD	Supplementary File 2
Occupation	3	What was their occupation at the time of the study?	Supplementary File 2
Gender	4	Was the researcher male or female?	Supplementary File 2
Experience and training	5	What experience or training did the researcher have?	Supplementary File 2
Relationship with participants			
Relationship established	6	Was a relationship established prior to study commencement?	3
Participant knowledge of the interviewer	7	What did the participants know about the researcher? e.g. personal goals, reasons for doing the research	3

Interviewer characteristics	8	What characteristics were reported about the inter viewer/facilitator? e.g. Bias, assumptions, reasons and interests in the research topic	Supplementary File 2
**Domain 2: Study design**			
Theoretical framework			
Methodological orientation and Theory	9	What methodological orientation was stated to underpin the study? e.g. grounded theory, discourse analysis, ethnography, phenomenology, content analysis	3
Participant selection			
Sampling	10	How were participants selected? e.g. purposive, convenience, consecutive, snowball	3
Method of approach	11	How were participants approached? e.g. face-to-face, telephone, mail, email	3
Sample size	12	How many participants were in the study?	3
Non-participation	13	How many people refused to participate or dropped out? Reasons?	3
Setting			
Setting of data collection	14	Where was the data collected? e.g. home, clinic, workplace	3
Presence of nonparticipants	15	Was anyone else present besides the participants and researchers?	3
Description of sample	16	What are the important characteristics of the sample? e.g. demographic data, date	4 (Table 1)
Data collection			
Interview guide	17	Were questions, prompts, guides provided by the authors? Was it pilot tested?	2
Repeat interviews	18	Were repeat interviews carried out? If yes, how many?	3
Audio/visual recording	19	Did the research use audio or visual recording to collect the data?	3
Field notes	20	Were field notes made during and/or after the interview or focus group?	3
Duration	21	What was the duration of the interviews or focus group?	3
Data saturation	22	Was data saturation discussed?	4 + 9
Transcripts returned	23	Were transcripts returned to participants for comment and/or correction?	3

Domain 3: analysis and findings			
Data analysis			
Number of data coders	24	How many data coders coded the data?	Supplementary File 2
Description of the coding tree	25	Did authors provide a description of the coding tree?	3 + Supplementary File 2
Derivation of themes	26	Were themes identified in advance or derived from the data?	3-4
Software	27	What software, if applicable, was used to manage the data?	3
Participant checking	28	Did participants provide feedback on the findings?	4
Reporting			
Quotations presented	29	Were participant quotations presented to illustrate the themes/findings? Was each quotation identified? e.g. participant number	4-7
Data and findings consistent	30	Was there consistency between the data presented and the findings?	4-7
Clarity of major themes	31	Were major themes clearly presented in the findings?	5
Clarity of minor themes	32	Is there a description of diverse cases or discussion of minor themes?	4-7

Developed from: Tong A, Sainsbury P, Craig J. Consolidated criteria for reporting qualitative research (COREQ): a 32-item checklist for interviews and focus groups. *International Journal for Quality in Health Care*. 2007. Volume 19, Number 6: pp. 349 – 357
