# Supplementary material for: Motivations and Limits for COVID-19 Policy Compliance in Germany and Switzerland
Source: Int J Health Policy Manag. 2021 Apr 21;11(8):1342–53. doi: 10.34172/ijhpm.2021.30 (PMC9808338; doi:10.34172/ijhpm.2021.30)
Supplement: Supplementary file 2 — Methods Additional Information. [file ijhpm-11-1342-s002.pdf]

Article title: Motivations and Limits for COVID-19 Policy Compliance in Germany and Switzerland

Journal name: International Journal of Health Policy and Management (IJHPM)

Authors' information: Bettina M. Zimmermann[1,2]*, Amelia Fiske[1], Stuart McLennan[1,2], Anna Sierawska[1], Nora Hangel[1]¶, Alena Buyx1¶

[1]Institute of History and Ethics in Medicine, Technical University of Munich, Munich, Germany.

[2]Institute for Biomedical Ethics, University of Basel, Basel, Switzerland.

(*corresponding author: bettina.zimmermann@tum.de)

¶ Authors 5 and 6 contributed equally to this paper and share last authorship.

Supplementary file 2: Methods Additional Information

Author contributions

Table S1: Overview of author contributions for data collection and analysis. All co-authors are trained in qualitative research methods.

	Germany	Switzerland
Data collection (interviewers)	BZ, AF, AS, NH, AB	BZ, NH
Development of Master Coding Scheme	BZ & NH as part of the SolPan consortium data analysis group	
Initial interview coding	BZ, AF, AS, NH	BZ, NH, SM
Interview coding double-check	BZ, NH, 3 student assistants	BZ, NH, 3 student assistants
Initial country-specific data analysis, memo writing (queries 1-3)	NH, 1 student assistant	BZ
Theme development, iterative data analysis (query 4)	BZ, NH, SM, AB, AF	

Author qualifications

Dr. phil. des. Bettina Zimmermann has an educational background in biomedical sciences and social sciences and a PhD in empirical bioethics. She is formally trained in qualitative research in the social sciences and has successfully conducted and published qualitative research in the field of bioethics.

Dr. Amelia Fiske is a cultural anthropologist, specializing in medical anthropology, science studies, and the environmental humanities. She has extensive qualitative research experience, including the completion of more than 27 months of ethnographic fieldwork for her PhD. She has expertise in in-depth, life history, and 'walking' interviews, as well as other bibliographic and participant-observation methodologies.

Dr. Stuart McLennan is an interdisciplinary bioethicist and received training in qualitative research methods during his PhD in empirical bioethics. He has extensive experience conducting and publishing qualitative research in the health care setting.

Anna Sierawska holds MA in philosophy, MSc in bioethics and MPhil in Applied Health Sciences. She completed a "Qualitative Research Methods" training led by the Health Experiences Research Group at the University of Oxford; she was a member of the interdisciplinary Qualitative Research Group at the University of Aberdeen where she also used to teach "ethics of qualitative research" for a Master of Public Health program. Anna has conducted interviews with various stakeholders, including healthcare professionals, members of the general population (also children), and experts.

Dr. Nora Hangel holds a PhD in philosophy. She has used qualitative research methods in the context of medical ethics since 2007 and specialized as a postdoc in empirical philosophy of science and science studies. She completed qualitative and mixed methods PhD courses at Aarhus University (BBS) in Denmark and has successfully conducted and published qualitative research in the fields of medical ethics, philosophy of science, and science studies.

Prof. Dr. Alena Buyx holds the chair of ethics in medicine and health technologies and is Director of the Institute of History and Ethics in Medicine at Technical University Munich. She is a medical doctor with postgraduate degrees in philosophy and sociology.

Atlas.ti queries

Query 1: (*solidarity | SUPPORTING/NOT supporting PRACTICES | supporting_not-family) & (perceiving_vulnerabilities_in others | MA_describing behav norms_shoud do/should not | MA_AGENCY MORAL agency | perceiving_a challenge | emotions_positive_by self | emotions_negative_by self)

Query 2: (rel_compliance_others | rel_non-compliance_self | rel_non-compliance_others | rel_compliance_self) & (MA_AGENCY MORAL agency | rel_disposition twd gov_criticising/doubt/skeptic | protecting_others | protecting_self | CHANGING PRACTICES | changing/adapt/rearrange_self | MA_expressing norms_general | MA_expressing moral position_specific | MA_describing behav norms_shoud do/should not)

Query 3: (perceiving_vulnerabilities_in others | *solidarity | supporting_not-family) & (MA_AGENCY MORAL agency | rel_disposition twd gov_criticising/doubt/skeptic | about the elderly_general/specific | rel_non-compliance_others | rel_non-compliance_self)

Query 4: rel_compliance_others | rel_compliance_self | rel_non-compliance_others | rel_non-compliance_self

Queries 1-3 were used for the explorative analysis, which was done separately for each country. Findings were then compared and combined for subsequent analysis. Query 4 was used to critically check preliminary findings for consistency and completeness.

Reflections about personal biases

The COVID-19 pandemic poses significant challenges not only for societies but also for individuals, including the co-authors. While conducting the interviews, the majority of co-authors had additional childcare duties due to closed childcare facilities and schools. Living with the pandemic, the researchers were constantly exposed to the ever-changing policy landscape and situation and experienced similar uncertainties as expressed by the interview participants. Views and attitudes were shaped particularly by the authors` country of residence (Switzerland for BZ, Germany for AF, SM, AS, NH, AB). This constant personal involvement made it difficult at times to look at the data and the policy context in an analytical manner. The research

team tried to stay reflective over these potential biases through frequent discussions and exchanges about the data, but also about personal attitudes of compliance and policies.
